# Clinical and virological outcomes with baloxavir compared with oseltamivir in pediatric patients aged 6 to < 12 years with influenza: an open-label, randomized, active-controlled trial protocol

**DOI:** 10.1186/s12879-021-06494-w

**Published:** 2021-08-09

**Authors:** Nobuhisa Ishiguro, Ichiro Morioka, Takashi Nakano, Masashi Furukawa, Shintaro Tanaka, Masahiro Kinoshita, Atsushi Manabe

**Affiliations:** 1 Division of Infection Control, Hokkaido University Hospital, Nishi 5, Kita 14, Kita-ku, Sapporo, 060-8648 Japan; 2 Department of Pediatrics and Child Health, Nihon University School of Medicine, Tokyo, Japan; 3 Department of Pediatrics, Kawasaki Medical School, Okayama, Japan; 4 Biostatistics Center, Shionogi & Co., Ltd., Osaka, Japan; 5 Medical Affairs, Shionogi & Co., Ltd., Tokyo, Japan; 6 Medical Affairs, Shionogi & Co., Ltd., Osaka, Japan; 7 Department of Pediatrics, Graduate School of Medicine, Hokkaido University, Sapporo, Japan

**Keywords:** Baloxavir, Child, Clinical trial protocol, Influenza, Japanese, Oseltamivir

## Abstract

**Background:**

Children with influenza virus infections are prone to complications and are common sources of influenza transmission. Baloxavir marboxil inhibits cap-dependent endonuclease and was approved for influenza treatment in adolescent, adult, and pediatric patients in Japan. The miniSTONE-2 study included pediatric patients with influenza (1 to < 12 years) and demonstrated similar median times to alleviation of signs and symptoms of influenza with a single dose of baloxavir granules (weight < 20 kg: 2 mg/kg, ≥ 20 kg: 40 mg) and oseltamivir. Although the baloxavir dose in miniSTONE-2 was higher than the Japanese-approved dose, baloxavir exposure in miniSTONE-2 was similar to Japanese pediatric patients who receive the Japanese-approved dose. This study will be the first randomized active-controlled study in pediatric patients with influenza using the Japanese-approved dose of baloxavir.

**Methods:**

This is a multicenter, open-label, randomized, active-controlled trial in which 200 Japanese subjects aged 6 to < 12 years with influenza virus infection are randomly allocated (2:1) to a single dose of baloxavir at the approved dose in Japan (weight ≥ 10 to < 20 kg: 10 mg, ≥ 20 to  < 40 kg: 20 mg, ≥ 40 kg: 40 mg) or oseltamivir twice daily for 5 days. The primary clinical endpoint is the time to illness alleviation of influenza, from administration of baloxavir or oseltamivir until the following criteria were met and sustained for at least 21.5 h (24 h—10%): cough and nasal discharge/nasal congestion rated as absent or mild axillary body temperature < 37.5 °C. The primary analysis population is the intention-to-treat infected population, which includes all pediatric subjects who receive at least one dose of study drug and have confirmed influenza virus infection by reverse transcription-polymerase chain reaction. The safety population includes all subjects who receive at least one dose of study drug.

**Discussion:**

No comparative studies have been conducted to confirm the efficacy and safety of baloxavir versus a comparator in pediatric patients with influenza infection in Japan. The outcomes from this trial will provide evidence on the efficacy and safety of baloxavir as an antiviral treatment option for Japanese pediatric patients with influenza infection. *Trial registration* Japan Registry of Clinical Trials: jRCTs011200011. Registered November 2020. (https://rctportal.niph.go.jp/en/).

## Background

Influenza virus infections commonly occur in children and can be associated with serious complications such as febrile seizures and influenza encephalopathy, and significant socioeconomic burden [1–4]. Globally, influenza infection is estimated to be responsible for 16% of hospitalizations for respiratory disease among children 5–17 years [3] and 5% of hospitalizations for acute lower respiratory disease among children < 5 years [5]. Similarly, in Japan, approximately 25% of influenza infections occur in children 6–12 years, and hospitalizations due to severe influenza-related complications are higher in children than adults and are highest in children < 5 years [6]. Preventing influenza infection in children is particularly important because they are a common source of influenza transmission among households and the wider community [7, 8]. While annual vaccination is the most effective means of preventing influenza and influenza-related complications [1], effectiveness is limited by the emergence of new influenza virus subtypes, the increased susceptibility of children to influenza infection, and decreased vaccine coverage and availability [9–11].

Neuraminidase inhibitors (NAIs) are recommended for the treatment of influenza virus infection in children who are critically ill or at high risk of complications, are symptomatic and have household members who are at high risk of developing influenza complications, and are within 2 days of symptom onset [1]. In children, NAIs have been shown to reduce the duration of influenza illness [12] and the incidence of influenza-associated acute otitis media [13], and may contribute to improved survival in those who are critically ill [14]. However, there may be differences in the effectiveness of NAIs across influenza types in young children (1–5 years) as shown by oseltamivir, which is less effective against influenza type B virus than influenza type A virus [15]. In addition to intrinsic factors such as age and immune status, other factors that may contribute to reduced effectiveness include nonadherence to a repeat-dose treatment regimen or difficulties with inhaled routes of administration [16, 17]. Further, emerging resistance to current NAIs indicates that new antiviral treatments with different mechanisms of action are needed to complement the currently available options for antiviral therapy [18, 19].

Baloxavir marboxil (hereafter termed baloxavir) is an oral antiviral prodrug in which the metabolite, baloxavir acid, selectively inhibits the cap-dependent endonuclease activity of the influenza type A and B polymerase acidic protein (PA) protein [20, 21]. In Japan, baloxavir is approved for the treatment of influenza in adolescents and adults, and in pediatric patients. In adolescents and adults aged 12–64 years with uncomplicated influenza, single-dose baloxavir significantly improves time to alleviation of influenza symptoms compared with placebo and significantly reduces infectious virus titer compared with placebo and oseltamivir (CAPSTONE-1) [22]. In addition, in high-risk adolescents and adults (12–89 years) with uncomplicated influenza, early treatment with baloxavir has been shown to reduce influenza-associated complications compared with placebo (CAPSTONE-2) and also significantly shorten the time to improvement of influenza symptoms compared with oseltamivir in patients with influenza type B [23]. The efficacy and safety of baloxavir 2% granules for oral suspension have been assessed in a double-blind, randomized, active-controlled trial in non-Japanese children aged 1– < 12 years with influenza (miniSTONE-2) [24]. In this study, a single dose of baloxavir granules (2 mg/kg for those weighing < 20 kg and 40 mg for those weighing ≥ 20 kg) was similarly effective at alleviating signs and symptoms of influenza compared with oral oseltamivir administered twice daily for 5 days, and baloxavir shortened the median time to cessation of viral shedding compared with oseltamivir. Although the dose of baloxavir in miniSTONE-2 was higher than the dose that is approved for children in Japan (10 mg for those weighing 10 to < 20 kg and 20 mg for those weighing 20 to < 40 kg), studies of baloxavir in Asian and non-Asian individuals [25, 26] suggest that baloxavir exposure in the miniSTONE-2 study was similar to Japanese individuals who are administered the Japanese approved dosage. Two open-label noncomparative studies in Japanese children have been conducted, which showed that a single weight-adjusted dose of baloxavir granules [27] or tablet (in accordance with the approved dose regimen) [28] is well tolerated and appears to be clinically and virologically effective. In addition, findings from a large postmarketing surveillance study enrolling more than 3000 patients in Japan showed that the median time to alleviation of influenza symptoms in the pediatric population aged 6 to < 12 years of age (n = 703), was similar to that in adults [29]. However, no randomized active-controlled studies comparing baloxavir with oseltamivir have been conducted in a Japanese pediatric population at the Japanese approved dose of baloxavir.

This open-label randomized controlled trial was designed to assess the clinical and virological efficacy and tolerability of single-dose baloxavir compared with oseltamivir twice daily for 5 days for the treatment of influenza infection in Japanese children aged 6 to < 12 years.

## Methods

### Trial design

This is a multicenter, open-label, randomized, active-controlled trial of the clinical and virological efficacy of a single oral dose of baloxavir compared with oral oseltamivir twice daily for 5 days in otherwise healthy subjects aged 6 to < 12 years with influenza during the 2020–2021 influenza season in Japan. The trial, which started in November 2020, will enroll patients from approximately 82 pediatric clinics or pediatric hospital departments in Japan.

The trial protocol was reviewed/approved by the Hokkaido University Certified Review Board (CRB; approval number 020-005, October 2020). The trial will adhere to the Clinical Trials Act (Act No. 16, April 14, 2017), the Declaration of Helsinki, and Ethical Guidelines for Medical and Health Research Involving Human Subjects in Japan, and has been registered at the Japan Registry of Clinical Trials (https://rctportal.niph.go.jp/en/; jRCTs011200011).

All pediatric subjects will receive adequate information about the nature, purpose, and possible risks and benefits of the trial and about alternative therapeutic choices via a parent or legal guardian who will be provided with an informed consent form approved by the CRB. Every effort will be made to obtain written informed assent from each patient in accordance with their level of comprehension. Written informed consent will be obtained from a parent/legal guardian for each enrolled subject and every effort will be made to obtain written informed assent from pediatric subjects according to their capabilities. A subject and a parent/legal guardian will be free to withdraw from the trial at any time upon request. An investigator may terminate a pediatric subject’s participation in the study if the subject meets a newly developed or not previously recognized exclusion criterion that precludes further participation.

**Fig. 1:**
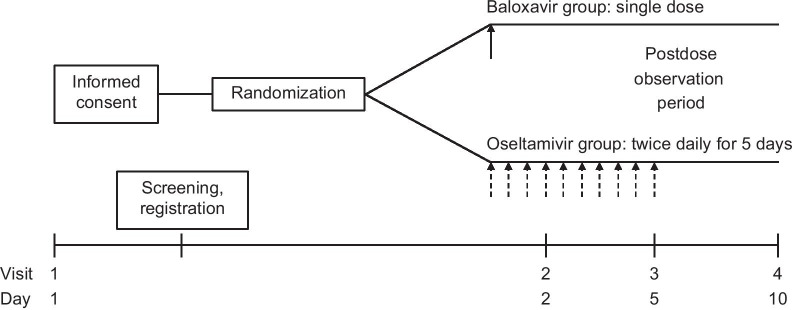
Trial timeline

Eligible subjects will be registered and randomly allocated (2:1) to the baloxavir and oseltamivir groups using an interactive web response system (Fig. 1). Randomization will be stratified using stochastic minimization to balance age (≥ 6 to < 9 years, ≥ 9 to < 12 years) and baseline total score for the Influenza Symptom Severity Scale for respiratory symptoms (cough and nasal discharge/nasal congestion) (≤ 3, ≥ 4). The Influenza Symptom Severity Scale is rated by subjects or with assistance from a parent/guardian on a 4-point scale (0 = no symptoms, 1 = mild, 2 = moderate, 3 = severe). All study drugs will be administered by the subjects themselves or with assistance from a parent/guardian.

Screen failures are defined as pediatric patients who do not meet the eligibility criteria. Reasons for discontinuation from the trial will include the following: decision to discontinue by a pediatric subject or their parent/legal guardian; death or any serious adverse event (SAE) or intolerance to study drug as determined by the investigator; a positive severe acute respiratory syndrome coronavirus 2 (SARS-CoV-2) test or suspected coronavirus disease 2019 (COVID-19); any repeated deviation from the protocol (e.g. repeated use of concomitant prohibited drugs) for the duration of the trial; or any other reason as determined by the investigator.

### Trial participants

Inclusion and exclusion criteria are described in full in Table 1. To be included in the trial, written informed consent from a parent/legal guardian will be required for each pediatric subject enrolled (every effort will also be made to gain written informed assent). In addition, pediatric subjects are to be otherwise healthy, aged 6 to < 12 years with a body weight ≥ 10 kg, have a positive rapid influenza diagnostic test which detects an antigen of influenza virus, and be judged by the investigator or sub-investigator to be able to take baloxavir tablet(s) and oseltamivir capsules or dry syrup. The main exclusion criteria are severe influenza symptoms requiring inpatient treatment, administration of systemic corticosteroids or immunosuppressive therapy, presence of an infectious disease requiring systemic antimicrobial therapy at the time of screening, allergies and/or a history of clinically problematic intolerance to anti-influenza drugs and/or acetaminophen, or a positive SARS-CoV-2 diagnostic test or suspected COVID-19 disease at the time of screening.

**Table 1:** Inclusion and exclusion criteria

Inclusion criteria	
Age 6 to < 12 years	
Body weight ≥ 10 kg	
Diagnosed with influenza infection defined as: Fever (axillary temperature ≥ 38 °C) A positive RIDT by nasopharyngeal swab, pharyngeal swab, or nasal discharge At least one moderate or severe respiratory symptom (cough and nasal discharge/nasal congestion)	
Onset of first confirmed fever (≥ 37.5 °C) no more than 48 h before screening (Day 1)	
Judged by the investigator as able to take baloxavir tablet(s) and oseltamivir capsules or dry syrup	
Where possible, written informed assent from children Written informed consent from a parent or legal guardian	
Parents/guardians willing and able to comply with the trial requirements Patients who can comply with the trial requirements to the level of their understanding	
Exclusion criteria	
Severe influenza symptoms requiring hospitalization	
Risk factors for influenza infection including: Chronic respiratory diseases including uncontrolled bronchial asthma Hepatitis, liver cirrhosis Neurological disorders and neurodevelopmental disorders including brain, spinal, peripheral, and muscle disorders Endocrine disorders including diabetes, thyroid disease, and adrenal gland abnormalities Heart disease requiring drug treatment including congenital heart disease, congestive heart failure, and coronary artery disease	
Receiving systemic corticosteroids or immunosuppressive therapy	
Primary immunodeficiency syndrome	
Severe renal impairment (creatinine clearance < 30 mL/min or eGFR < 30 mL/min/1.73 m^2^)	
Human immunodeficiency virus infection	
Malignant disease within the past 5 years	
Disorders of consciousness including abnormal behavior^a^ and convulsions, and complicated by encephalitis/encephalopathy	
History of encephalitis/encephalopathy or epilepsy not controlled with antiepileptic drugs or abnormal behavior^a^ associated with influenza infection in the past 2 years	
Infectious diseases requiring systemic antimicrobial therapy and/or antiviral medicine (except anti-influenza medicine) at screening	
Considered by the investigator to be using prohibited drugs	
Receiving baloxavir, amantadine, or NAIs (oseltamivir, zanamivir, peramivir, and laninamivir) within 30 days before screening	
History of allergies to anti-influenza drugs, or acetaminophen, or clinically problematic intolerance	
Positive SARS-CoV-2 or suspected COVID-19 disease	
Any underlying disease that may affect study outcomes	
Difficulty collecting blood from a vein Patients received an investigational or unapproved drug within 30 days or 5 half-lives, whichever is longer, before screening Patients with diseases or conditions which, in the opinion of the investigator, may make it difficult to ensure patient safety or study data quality Patients who, in the opinion of the investigator, are inappropriate for participation in the study Female patients who has experienced menarche and who are pregnant, plan to become pregnant during the study period, or are breastfeeding	

^a^Actions such as a sudden start, jump, or any other unexpected behavior that may be life-threatening

*COVID-19* coronavirus disease 2019; *eGFR* estimated glomerular filtration rate; *NAI* neuraminidase inhibitor; *RIDT* rapid influenza diagnostic test; *SARS-CoV-2* severe acute respiratory syndrome coronavirus 2

### Treatment protocol

Eligible pediatric subjects will receive a single oral dose of baloxavir tablet at the approved dose in Japan (10 mg for children weighing ≥ 10 to < 20 kg, 20 mg for ≥ 20 to < 40 kg, and 40 mg for ≥ 40 kg) [30] on Day 1 or oral oseltamivir capsules or dry syrup (2 mg/kg for subjects weighing < 37.5 kg, 75 mg for ≥ 37.5 kg) twice daily on Days 1–5 (Fig. 1). If subjects skip a dose, the dose will be taken as soon as they are aware of missing a dose unless the next dose is within 2 h. The subjects will be allowed to use acetaminophen (≤ 60 mg/kg/day or < 1500 mg/day, which is the maximum dose for adults) as needed throughout the trial for the alleviation of severe symptoms. Pediatric subjects or their parent/guardian will record the date and time of the use of acetaminophen using an electronic patient-reported outcome (ePRO) diary.

The following drugs or treatments will be prohibited during the trial: systemic antiviral drugs, antibacterial or antifungal drugs (except for suspected bacterial or fungal infection after administration of study drug), antipyretic analgesics (except acetaminophen), injection or oral corticosteroids (inhalant is acceptable), immunosuppressive drugs, and warfarin.

### Clinical and laboratory monitoring

Eligible subjects will undergo a confirmatory test for influenza A or B at the screening visit on Day 1. To be included in the primary analysis population, influenza virus infection will be confirmed by reverse transcription-polymerase chain reaction (RT-PCR). A full description of the visit and assessment schedule is provided in Table 2. The following outcome measures will be assessed by subjects or their parent/guardian and recorded in an ePRO diary from postdose Day 1 to postdose Day 9: influenza symptom severity and body temperature (axillary) using an electronic thermometer taken right before or at least 4 h after administration of acetaminohen, if relevant; the severity of seven influenza symptoms (cough, sore throat, headache, nasal discharge/nasal congestion, feverish or chills, muscle ache or joint pain, and fatigue) will be assessed by subjects ≥ 7 years who are deemed able to assess influenza symptoms themselves; the severity of two influenza symptoms (cough, nasal discharge/nasal congestion) will be assessed by a parent/guardian for subjects < 7 years and/or those ≥ 7 years who are considered by the investigators to not able to assess influenza symptoms themselves.

**Table 2:** Visit and assessment schedule

Day	1		2 +1 day	3	4	5 -1 to +2 days	6	7	8	9	10 −1 to +4 days 4	Discontinuation +14 days
Visit	1^a^		2			3						
	Pre dose	Post dose										
Informed consent	X											
Inclusion/exclusion criteria	X											
Demographics, clinical characteristics, and medical history	X											
Enrollment and randomization	X											
Baloxavir administration		X										
Oseltamivir administration		X	X	X	X	X						
Diary (ePRO)												
Body temperature	X	X^a^	qid		bid							
Influenza symptom severity	X	X^a^	bid									
Medical examination^b^	X		X			X					X	X
Vital signs	X		X			X					X	X
Influenza RIDT	X^c^											
SARS-CoV-2 test	X^d^		X^e^									
Influenza virus tests (RT-PCR, titer, RNA, PA, or NA sequence)	X		X			X					X	X
Influenza virus serum antibody titer test (HI method)	X											
Intrahousehold influenza infection rate questionnaire											X	X
TEAE reporting	X	X	X	X	X	X	X	X	X	X	X	X

X denotes assessment to be undertaken

*bid* twice a day; *COVID-19* coronavirus disease 2019; *ePRO* electronic patient-reported outcome; *HI* hemagglutination inhibition; *NA* neuraminidase; *PA* polymerase acidic protein; *qid* four times a day; *RIDT* rapid influenza diagnostic test; *RT-PCR* reverse transcription-polymerase chain reaction; *SARS-CoV-2* severe acute respiratory syndrome coronavirus 2; *TEAE* treatment-emergent adverse event

^a^Not measured if study drug taken after 18:00

^b^Participants’ height and body weight are measured on Day 1 (predose/screening)

^c^Participants who have an RIDT and/or other influenza virus test within 48 h of symptom onset but before written informed consent do not need to take an RIDT and/or other influenza virus test on Day 1

^d^Participants who are determined to be SARS-CoV-2 negative according to the COVID-19 clinical guideline in Japan (https://www.mhlw.go.jp/content/000712473.pdf) before giving written consent do not need to undergo a SARS-CoV-2 test on Day 1

^e^This test will be conducted according to the COVID-19 clinical guideline as appropriate

The presence of SARS-CoV-2 infection will be assessed using an approved rapid SARS-CoV-2 test which includes but not limited to SARS-CoV-2 antigen test. For influenza virology tests, nasopharyngeal swabs will be tested for influenza virus type/subtype, viral RNA load and infectious influenza virus titer, and amino-acid substitutions in the PA protein subunit and neuraminidase (NA) using Sanger sequencing and a nested PCR (LSI Medience Corporation, Tokyo, Japan), as previously described [27]. Medical examinations will be conducted by investigators, and vital signs will be measured by investigators, nurses, or medical technicians, as appropriate.

For the intrahousehold (cohabitants or family members who live together) influenza virus transmission rate, household members who give their written consent will be provided with a paper questionnaire on Day 1 (predose). Completed questionnaires will be collected on Day 10. The questionnaire covers information on the following: household size (including age category); the number of household members infected from the pediatric subject’s enrollment up to Day 10 or until discontinuation from the study; and the date of each household member’s diagnosis of influenza infection, use of anti-influenza drug therapies, and the date of their recovery of normal body temperature (< 37 °C).

### Outcome measures and endpoints

The primary clinical endpoint is the time to illness alleviation (TTIA), defined as the time from baloxavir or oseltamivir administration until the following criteria were reached and sustained for at least 21.5 h (24 h—10%): both cough and nasal discharge/nasal congestion assessed as 0 (absent) or 1 (mild) and axillary temperature less than 37.5 °C (Table 3).

**Table 3:** Outcome measures and endpoints

Efficacy	
Primary clinical endpoints	
Time to illness alleviation of influenza, defined as the time from baloxavir or oseltamivir administration until the following criteria were reached and sustained for at least 21.5 h (24 h—10%): both cough and nasal discharge/nasal congestion assessed as 0 (absent) or 1 (mild) and axillary temperature less than 37.5 °C	
Secondary endpoints	
Time to sustained resolution of influenza symptoms (cough, nasal discharge/nasal congestion) rated using the Influenza Symptom Severity Scale as absent (grade 0) or mild (grade 1) and resolution of fever (normal body temperature < 37.5 °C) for at least 72 h	
Time to resolution of fever (< 37.5 °C) for at least 12 h	
Time to sustained resolution of fever for at least 72 h	
Proportion of subjects with resolution of fever at each time point	
Time to resolution of all seven influenza symptoms (cough, sore throat, headache, nasal discharge/nasal congestion, feverish or chills, muscle ache or joint pain, and fatigue), rated using the Influenza Symptom Severity Scale as absent (grade 0) or mild (grade 1) for at least 21.5 h	
Time to resolution of each influenza symptom for at least 21.5 h	
Incidence of influenza-related complications (pneumonia determined by X-ray, bronchitis, sinusitis, otitis media)	
Incidence of influenza-related complications seen particularly in children (influenza-associated encephalitis or encephalopathy, febrile seizures, myositis) Incidence of death and hospitalization from influenza-related complications or influenza-related complications seen particularly in children	
Use of antibiotics	
Virological endpoints Infectious influenza virus titer and the amount of virus RNA (determined by RT-PCR) at each time point Change from baseline in infectious influenza virus titer and amount of virus RNA (determined by RT-PCR) at each time point Proportion of participants whose influenza virus titer and quantitative RT-PCR are above and below the lower limits of quantification at each time point Time to the first cessation of viral shedding based on the influenza virus titer, defined as the time postdose until virus titers reach the lower limit of quantification for the first time Time to sustained cessation of viral shedding based on influenza virus titer, defined as the time from the last positive virus titer postdose to the first virus titer that is below the lower limit of quantification	
Exploratory endpoints	
Emergence of amino-acid substitutions (PA/I38X and NA/H275Y) in subjects who are RT-PCR positive for influenza infection pre- and postdose. Treatment-emergent influenza virus substitutions in PA/I38 or NA/H275 will be defined as amino-acid substitution occurring between Day 1 and the last time point where the influenza virus RT-PCR cycle threshold is < 31 for the H1 subtype or < 29 for the H3 subtype Rate of intrahousehold influenza infection	
Safety	
AEs, AEs possibly related to baloxavir or oseltamivir, SAEs, vital signs	

*AE* adverse event; *NA* neuraminidase; *PA* polymerase acidic protein; *RT-PCR* reverse transcription-polymerase chain reaction; *SAE* serious adverse event

The main secondary endpoints include the time to sustained resolution of influenza symptoms (cough, nasal discharge/nasal congestion, and body temperature) for at least 72 h; the time to resolution of fever (normal body temperature < 37.5 °C) for at least 12 h; the time to sustained resolution of fever for at least 72 h; the proportion of subjects with resolution of fever at each time point; the time to resolution of seven influenza symptoms (cough, sore throat, headache, nasal discharge/nasal congestion, feverish or chills, muscle ache or joint pain, and fatigue) for at least 21.5 h; the incidence of influenza-related complications and those influenza-related complications seen particularly in children (influenza-associated encephalitis or encephalopathy, febrile seizures, myositis); the use of antibiotics for treatment of infectious disease secondary to influenza infection; and the change from baseline in infectious influenza virus titer.

The assessment of the safety and tolerability of study drugs will include the frequency and nature of adverse events (AEs), AEs related to study drugs, SAEs, and vital signs. All AEs will be managed in accordance with good medical practice by the participating physicians as appropriate. An AE will be considered “serious” if it results in death, is life-threatening, requires hospitalization or prolongation of hospitalization, or is persistent or results in significant incapacity or substantial disruption to daily life. All deaths and immediately life-threatening events, whether related or unrelated to study drug, will be reported to the CRB as soon as possible. All SAEs will be shared with all investigators participating in the study. If there is a reasonable possibility that the study procedure causes an unanticipated SAE, the sponsor of the study will report the SAE to the Pharmaceuticals and Medical Devices Agency in Japan.

### Statistical analysis

The planned sample size is 200 pediatric subjects with influenza (134 in the baloxavir treatment group and 66 in the oseltamivir treatment group). Because of the anticipated challenges in enrolling patients during the COVID-19 pandemic and because the study is not designed to demonstrate noninferiority or superiority considering feasibility, no formal statistical sample-size calculations will be performed. The intention-to-treat infected population for efficacy will include all subjects who receive at least one dose of baloxavir or oseltamivir and who have a confirmed diagnosis of influenza virus infection by RT-PCR on Day 1. Based on our past experience in clinical studies in Japan [27, 28], it is expected that almost all subjects with a positive rapid influenza diagnostic test be confirmed for influenza by RT-PCR. The safety population will consist of all subjects who receive at least one dose of baloxavir or oseltamivir. Statistical analysis will be based on descriptive statistics and no statistical tests will be used.

For the primary endpoint, the median TTIA (95% confidence interval [CI]) will be assessed using Kaplan–Meier curves for each treatment group, and the efficacy of baloxavir compared with oseltamivir will be assessed using between-group differences (95% CI). As a secondary analysis, the restricted mean survival time (RMST) and 95% CI up to Day 9 will be estimated for each treatment group by direct integration of the Kaplan–Meier survival curve. The between-group difference in RMST (95% CI) will also be calculated. The secondary time-to-event efficacy analyses will be assessed in the same manner as the primary analysis for the primary endpoint. For all other analyses, quantitative data will be summarized by descriptive statistics (mean, standard deviation [SD], maximum, median, minimum) and the mean between-group difference (95% CI). Qualitative data will be summarized by frequency, proportion, and the between-group difference in proportion (95% CI) using the Santner and Snell method.

The number of participants with AEs, SAEs, AEs leading to discontinuation, and AEs possibly related to study drug, as well as the number of deaths, will be summarized for each treatment group, and AEs will be coded using the Medical Dictionary for Regulatory Activities. Incidence rates will be calculated as the proportion of participants in the analysis population, and 95% CI using the Clopper-Pearson method. The change in each vital sign from baseline (predose) will be calculated for each treatment group.

Data obtained outside the allowable ranges described in Table 2 will be treated as missing, and values will not be imputed at each visit. All analyses will be conducted using SAS® software, version 9.4 (SAS Institute Inc., Cary, NC, USA).

### Data management and monitoring

The data collected will be transcribed onto an electronic Case Report Form maintained by EP‐CRSU Co., Ltd., Tokyo, Japan. Site monitoring visits will involve source data verification by MEDICEO Co., Ltd., Tokyo for 2020 and Falfield Co., Ltd., Tokyo, Japan for 2021. Submitted data will be anonymized, reviewed for completeness and consistency, and then entered into a database. Data will be stored securely against unauthorized manipulation and accidental loss.

### Data security

The participants’ names and other confidential information will be secured by medical confidentiality rules and will be treated according to the Act on the Protection of Personal Information in Japan.

### Trial status

This trial was declared and registered on November 20, 2020. Recruitment into the trial started in November 2020 and will end in April 2021 or until a total of 200 participants have been recruited.

## Discussion

MiniSTONE-2 was the first double-blind, randomized, active-controlled trial that was specifically designed to compare the efficacy and safety of baloxavir with oseltamivir in children aged 1 to < 12 years [24]. In that study, the median time (95% CI) to alleviation of influenza symptoms was similar between the treatment groups (baloxavir: 138.1 [116.6–163.2] hours versus oseltamivir: 150.0 [115.0–165.7] hours). In addition, and similar to the findings in adolescents and adults [22, 23], the reduction in viral load was faster with baloxavir compared with oseltamivir, with the median time to cessation of viral shedding shortened by approximately 24 h with baloxavir [24]. Consistent with the findings from studies in otherwise healthy adolescents and adults and those at high risk of influenza-associated complications, baloxavir was well tolerated with no unexpected AEs reported [22, 23], and the most frequently reported AEs were gastrointestinal (vomiting/diarrhea) in both treatment groups [24]. Although miniSTONE-2 was the first randomized active-controlled trial of baloxavir in children, this study was conducted in a non-Japanese population and included baloxavir 2% granules for oral suspension rather than tablets. In addition, in miniSTONE-2, baloxavir was administered at a different dose than is approved for use in Japan, but baloxavir exposure in miniSTONE-2 study is expected to be similar to that of Japanese individuals who are administered the Japanese approved dosage [25, 26].

The present study will be one of the first to assess treatment-emergent amino-acid substitutions for both PA and NA among children treated with baloxavir and oseltamivir in the same study. Several studies suggest that treatment-emergent antiviral resistance is more likely to emerge in children than in adults [27, 28, 31, 32], which is of concern because of the potential risk of transmission of virus variants to close contacts [33]. The frequency of treatment-emergent PA/I38X amino-acid substitutions that are associated with reduced susceptibility to baloxavir has been shown to be more than twofold higher in children than in adults [27, 28]. Findings from a global 7-year surveillance study showed that treatment-emergent resistance to oseltamivir (NA/H275Y for N1 viruses and NA/R292K for N2 viruses) is highest among young children (1–5 years) compared with adults and older children or adolescents [31, 32]. Although it has not been established why young children may be more susceptible to the emergence of antiviral treatment resistance, it is thought that influenza virus variants emerge in response to selective pressure during drug treatment, possibly as a result of the immature immune response in children, at a time when drug concentrations in plasma have started to wane [27, 28, 32].

Findings from the two open-label noncomparative studies of weight-adjusted baloxavir suggest that baloxavir is likely to be clinically effective compared with NAIs in Japanese children aged 6 to < 12 years [27, 28]. In both studies, there was a rapid reduction in virus titer, and the median TTIA was 45 h. Gastrointestinal events were the most commonly reported AEs in both studies, which is consistent with the findings in non-Japanese children in miniSTONE-2 [24] and with the findings from a large postmarketing surveillance study of baloxavir in Japan that included 896 children aged < 12 years [29]. In this surveillance study, adverse drug reactions occurred more frequently in children than in adults, but diarrhea was the most common adverse reaction in all age groups. Although these real-world studies provide valuable information on the use of baloxavir in Japan, no clinical studies have been conducted to confirm the effectiveness of baloxavir against a comparator in children (6 to < 12 years) with influenza infection in Japan.

This study will be the first randomized active-controlled trial to compare baloxavir and oseltamivir in Japanese children aged 6 to < 12 years with influenza virus infection. Although the planned sample size is largely based on the feasibility of conducting the trial during the COVID-19 pandemic, the planned number of subjects is higher than previous studies of baloxavir in Japanese children. Overall, this trial will extend the evidence on the efficacy and safety of baloxavir as an antiviral treatment option for Japanese children with influenza infection.

## Data Availability

Not applicable.

## Abbreviations

AE: Adverse event
bid: Twice a day
CI: Confidence interval
COVID-19: Coronavirus disease 2019
CRB: Certified Review Board
eGFR: Estimated glomerular filtration rate
ePRO: Electronic patient-reported outcome
HI: Hemagglutination inhibition
jRCT: Japan Registry of Clinical Trials
NA: Neuraminidase
NAI: Neuraminidase inhibitor
PA: polymerase acidic protein
qid: Four times a day
RIDT: Rapid influenza diagnostic test
RMST: Restricted mean survival time
RT-PCR: Reverse transcription-polymerase chain reaction
SAE: Serious adverse event
SARS-CoV-2: Severe acute respiratory syndrome coronavirus 2
SD: Standard deviation
TEAE: Treatment-emergent adverse event
TTIA: Time to illness alleviation
